# Emotion Regulation and Complex Brain Networks: Association Between Expressive Suppression and Efficiency in the Fronto-Parietal Network and Default-Mode Network

**DOI:** 10.3389/fnhum.2018.00070

**Published:** 2018-03-16

**Authors:** Junhao Pan, Liying Zhan, ChuanLin Hu, Junkai Yang, Cong Wang, Li Gu, Shengqi Zhong, Yingyu Huang, Qian Wu, Xiaolin Xie, Qijin Chen, Hui Zhou, Miner Huang, Xiang Wu

**Affiliations:** Department of Psychology, Sun Yat-sen University, Guangzhou, China

**Keywords:** DMN, emotion regulation, FPN, graph theory, resting-state fMRI

## Abstract

Emotion regulation (ER) refers to the “implementation of a conscious or non-conscious goal to start, stop or otherwise modulate the trajectory of an emotion” (Etkin et al., 2015). Whereas multiple brain areas have been found to be involved in ER, relatively little is known about whether and how ER is associated with the global functioning of brain networks. Recent advances in brain connectivity research using graph-theory based analysis have shown that the brain can be organized into complex networks composed of functionally or structurally connected brain areas. Global efficiency is one graphic metric indicating the efficiency of information exchange among brain areas and is utilized to measure global functioning of brain networks. The present study examined the relationship between trait measures of ER (expressive suppression (ES) and cognitive reappraisal (CR)) and global efficiency in resting-state functional brain networks (the whole brain network and ten predefined networks) using structural equation modeling (SEM). The results showed that ES was reliably associated with efficiency in the fronto-parietal network and default-mode network. The finding advances the understanding of neural substrates of ER, revealing the relationship between ES and efficient organization of brain networks.

## Introduction

Emotion regulation (ER) refers to “the processes that influence which emotions we have, when we have them, and how we experience and express them” (Gross, 1998). People adopt a wide variety of ER strategies, such as situation selection, situation modification, attentional deployment, cognitive change and response modulation (Gross, 1998). In face of the complexity of ER, Gross and John (2003) suggested to focus on a smaller number of well-defined strategies: “Our focus on two specific, well-defined processes is predicated on the belief that our understanding of complex emotion regulatory processes is best advanced if we focus intensively on one or two processes at a time”. The two major ER strategies or forms as suggested by Gross and John (2003) are expressive suppression (ES) and cognitive reappraisal (CR). ES refers to the alteration of one’s response to an emotional incident, while CR refers to the change of how one views the emotional incident in order to alter one’s feelings. For example, when feeling sad after a heart-broken breakup, one could withhold any sad expression in order to control the intense feelings (i.e., ES); or he/she could persuade himself into thinking that the finished relationship is perhaps actually good for both (i.e., CR). Using ER tasks that typically instruct participants to use the ES or CR strategy when presented with emotionally negative stimuli or adopting the ER questionnaire (ERQ) that was specifically developed to assess the habitual ES and CR usage (Gross and John, 2003), CR has been shown to produce affective, cognitive and social consequences that are more beneficial, whereas ES has been consistently linked to more detrimental consequences such as depressive symptoms (Gross, 2002; Gross and John, 2003; Goldin et al., 2008).

Neural mechanisms of ER have been under investigation using brain-imaging approaches. Task-based functional magnetic resonance imaging (fMRI) studies have shown the involvement of the ventrolateral prefrontal cortex (vlPFC), inferior frontal gyrus (IFG), insula and amygdala in ES (Goldin et al., 2008; Lee et al., 2008) and the engagement of the dorsomedial PFC (dmPFC), dorsolateral PFC (dlPFC), vlPFC, insula, temporal cortex, parietal cortex and amygdala in CR (Kalisch, 2009; Diekhof et al., 2011; Buhle et al., 2014; Gross, 2015). Using the ERQ, studies analyzing gray matter volume and surface thickness have shown that brain structural variations in the ventromedial PFC (vmPFC), dorsal anterior cingulate cortex (dACC), dmPFC, superior frontal gyrus (SFG) and insula are associated with ES (Welborn et al., 2009; Giuliani et al., 2011a; Hermann et al., 2014; Wang et al., 2017), and CR is related to structural variations in the superior frontal cortex (SFC), vmPFC, dACC and amygdala (Welborn et al., 2009; Giuliani et al., 2011b; Hermann et al., 2014; Moore et al., 2016). Moreover, using the ERQ and resting-based fMRI, Wang et al. (2017) found that functional connections between the SFG and regions including the medial PFC (mPFC), precuneus and parahippocampal gyrus were related to ES gender difference.

Previous brain-imaging findings thus suggest the involvement of multiple brain areas in ER and indicate that ER may be related to the functioning of brain networks, which are composed of structurally or functionally connected brain regions (van den Heuvel and Hulshoff Pol, 2010; Smith et al., 2013). Whereas prior studies have investigated functions of individual brain regions in ER, it remains unknown whether and how ER is supported by global functioning of brain networks. To this end, we used global efficiency, one of the graph theory’s global metrics, to assess the role of global functioning of brain networks in ER. There has been a growing interest to investigate the structural and functional organization of the brain by graph-theory based analyses, which consider the brain as organized into complex networks consisting of nodes (brain regions) and edges (structural or functional connectivity between regions; Rubinov and Sporns, 2010; De Vico Fallani et al., 2014; Mears and Pollard, 2016). The topological properties of complex networks can be assessed by a variety of measures (for reviews, see Bullmore and Sporns, 2009; Rubinov and Sporns, 2010; Wang et al., 2010; De Vico Fallani et al., 2014), among which network efficiency is considered to be a “more biologically relevant metric” describing networks in terms of information exchange among brain regions (Wang et al., 2010). Network efficiency can be measured by global efficiency and local efficiency at the global and local level, respectively (Wang et al., 2010; De Vico Fallani et al., 2014; Stanley et al., 2015). Note that global efficiency is inversely related to path length and is suggested to be “easier to estimate than path length when studying sparse networks” (Bullmore and Sporns, 2009); and local efficiency is positively associated with clustering coefficient. Given the purpose of investigating global functioning of brain networks, the present study focused on the metric of global efficiency, which is defined as the average inverse shortest path length in the network (Beaty et al., 2016). Global efficiency has been found to be related to cognitive (e.g., intelligence (Li et al., 2009; van den Heuvel et al., 2009), working memory (Alavash et al., 2015; Stanley et al., 2015)) and social (e.g., personality trait (Beaty et al., 2016) functions, as well as mental disorders (e.g., major depressive disorder (Meng et al., 2014), bipolar disorder (Collin et al., 2016), attention deficit/hyperactivity disorder (Wang et al., 2009)).

Global efficiency can be analyzed for the whole brain network (Li et al., 2009; Wang et al., 2009; Meng et al., 2014; Stanley et al., 2015) or subnetworks (Sheffield et al., 2015; Beaty et al., 2016). For subnetworks, research of resting-state functional connectivity has shown that the brain can be organized into networks composed of functionally connected brain regions (van den Heuvel and Hulshoff Pol, 2010; Power et al., 2011; Raichle, 2011; Cole et al., 2013; Smith et al., 2013). In brain connectivity research the brain is usually divided into a set of non-overlapping regions and the definition of brain networks is related to the brain parcellation (note that a validated parcellation strategy is still lacking; Power et al., 2011; Wig et al., 2011). Power et al. (2011) divided the brain into 264 putative regions and showed that 13 networks based on the 264 regions are in good agreement with major functional systems of the brain. Cole et al. (2013) further reduced the 13 networks into 10 networks by excluding two networks with less clear functionality and combing the “hand” and “face” networks based on consensus of a unified primary motor system. The 10 networks based on the 264-region parcellation were the somato-motor network (SMN), cingulo-opercular network (CON), auditory network (Aud.), default mode network (DMN), visual network (Vis.), fronto-parietal network (FPN), salience network (SAN), subcortical network (Sub.), ventral attention network (VAN) and dorsal attention network (DAN); and have been widely adopted in studies investigating relationships between network properties and cognitive functions (Cole et al., 2014b; Thompson and Fransson, 2015; Uddin, 2015; Vatansever et al., 2015; Long et al., 2016). For the major brain areas that have been found to be involved in ER, the lateral PFC is a core region of the FPN (Cole et al., 2013), the media PFC is a key region of the DMN (Buckner et al., 2008; Broyd et al., 2009), and the ACC is an important component of the CON (Power et al., 2011; Sadaghiani and D’Esposito, 2015). Based on the relationships between individual brain areas and ER and the relationships between individual brain areas and networks, it could be indicated that global efficiency in the FPN, DMN and CON is more likely to be associated with ER. This inference, however, remains to be verified by experimental data and would be difficult to be considered as a strong prior hypothesis. For example, a study examining the Big Five personality traits showed that extraversion and agreeableness were correlated with activities in the midline regions of the DMN and neuroticism, openness and conscientiousness were correlated with activities in the parietal regions of the DMN (Sampaio et al., 2014). However, Beaty et al. (2016) found that only openness was associated with global efficiency in the DMN and suggested that the involvement of a brain region in a cognitive process does not necessarily imply the engagement of global functioning of the network to which that brain region belongs, because individual regions “are related to a wide range of cognitive, behavioral and emotional variables”.

Taken together, the present study aimed to examine the relationship between ER and global functioning of brain networks. The ERQ was used to assess trait measures of the two ER strategies, ES and CR (Gross and John, 2003; Wang et al., 2007). Graph theory-based methods were applied to resting-state fMRI data (Bullmore and Sporns, 2009), and global functioning of the whole brain network and the 10 predefined networks (Cole et al., 2013) was examined by analyzing global efficiency. The association between the ER strategies and global efficiency in the brain networks was evaluated using structural equation modeling (SEM) that models error variance separately from true measurement variance (Beaty et al., 2016). We hypothesized that ER would be associated with global functioning of brain networks as indexed by global efficiency. More specifically, we inferred that global efficiency in the FPN, DMN and CON is likely to be associated with ER. In addition to the FPN, DMN and CON, the whole brain network and other networks in the 10 predefined networks were also analyzed, with the purpose of a more comprehensive understanding of the relationship between global efficiency in networks and ER.

## Materials and Methods

### Participants

The present study recruited 54 participants from Sun Yat-sen University. Two participants were excluded for excessive motion in the MRI scan and four participants were excluded for missing behavioral data, and finally 48 participants (all right-handed, 13 male, mean age ± SD 22.77 ± 1.59) were included in the subsequent analyses. All participants were healthy and reported no history of neurological or psychiatric disorders, or cognitive or affective impairments. This study was carried out in accordance with the recommendations of research protocol approved by the Institutional Review Board of Psychology Department of Sun Yat-sen University with written informed consent from all subjects. All subjects gave written informed consent in accordance with the Declaration of Helsinki. The protocol was approved by the Institutional Review Board of Psychology Department of Sun Yat-sen University.

### MRI Data Acquisition and Preprocessing

The participants were scanned using a Siemens 3.0 Tesla MRI scanner (Siemens, Erlangen, Germany) located at South China Normal University (Guangzhou, China). Whole brain T2*-weighted resting-state functional images were acquired for 8 min as participants relaxed (stayed awake without thinking anything) with eyes closed, using an echo-planar imaging (EPI) sequence: repetition time (TR) = 2000 ms, echo time (TE) = 30 ms, flip angle (FA) = 90°, field of view (FOV) = 224 × 224 mm^2^, slices = 32, matrix = 64 × 64, slice thickness = 3.5 mm, voxel size = 3.5 × 3.5 × 3.5 mm^3^, 240 volumes, and interleaved slice ordering. Whole brain T1-weighted structural images were obtained in a sagittal orientation using the magnetization-prepared rapid gradient-echo (MPRAGE) sequence: TR = 2300 ms, TE = 3.24 ms, FA = 9°, FOV = 256 × 256 mm^2^, inversion time = 900 ms, matrix = 256 × 256, slices = 176, slice thickness = 1 mm, and voxel size = 1 × 1 × 1 mm^3^.

The data were preprocessed using Statistical Parametric Mapping (SPM12^1^) and Data Processing Assistant for Resting-State fMRI (DPARSF; Yang and Zang, 2010). Preprocessing consisted of standard resting-state functional connectivity preprocessing procedures as implemented in DPARSF, including removing the first 10 volumes of functional images, slice timing correction, motion correction (data of two participants were excluded under the criterion of 2 mm displacement or 2° rotation in any direction), coregistration of structure images to functional images, segmentation with the DARTEL method (Ashburner, 2007), normalization to the standard MNI space with the DARTEL method and resampling functional images at a voxel size of 3 × 3 × 3 mm^3^, removing linear trends, regressing out nuisance variables (head motion parameters, white matter signals, and cerebrospinal fluid signals), filtering (0.01–0.1 Hz), and spatial smoothing (4-mm FHWM). Following previous work analyzing functional connectivity using graphic approaches (Zhao et al., 2012; Cole et al., 2013; Arnold et al., 2014; Santarnecchi et al., 2014; Beaty et al., 2016), in the present study whole brain signal was not regressed out as a nuisance variable because of the current controversy over global signal regression (Murphy et al., 2009) and the potential impact of global signal removal on topological properties of brain networks (Santarnecchi et al., 2014).

### Network Construction and Graphic Analyses

Graph analyses were conducted using graph theoretical network analysis (GRETNA; Wang et al., 2015). The present study adopted the 10 predefined networks based on the parcellation of the brain with 264 cortical and subcortical 10-mm diameter spherical regions (for detailed information, see Power et al., 2011; Cole et al., 2013). For each of the 10 networks in each participant’s dataset, regional time series were calculated by averaging voxel time series in each of the N regions in that network, and a N × N functional connectivity matrix was constructed by computing the Pearson correlation (with Fisher transformation) between each pair of regional time series of the N regions. The brain regions represented network nodes and the correlations between nodes represented network edges. The adjacency matrix was then computed from the correlation matrix by applying a threshold, which resulted in the binary undirected graph. Note that different thresholds would generate graphs of different connection density or sparsity, and network properties are suggested to be examined over a wide range of thresholds since currently there is no “good” threshold for graphic analysis (Bullmore and Sporns, 2009; Langer et al., 2012; Power et al., 2013). Therefore network thresholding in the present study used Fisher transformed *r* values ranging from 0.15 to 0.85 (Achard et al., 2006; He et al., 2007; Langer et al., 2012; Arnold et al., 2014; Santarnecchi et al., 2014), with the aim of a more comprehensive analysis. Moreover, whether a network would have the property of the small world was evaluated for each threshold. The small-world topology is characterized through the characteristic path length *L* and clustering coefficient *C* (Watts and Strogatz, 1998). The characteristic path length is defined as the average of the shortest path lengths between any pair of nodes in the network. The clustering coefficient is defined as the average of the clustering coefficients over all nodes, where the clustering coefficient of a node is defined as the proportion of possible connections that actually exist between the nearest neighbors of a node. Compared with random networks that have the same number of nodes, same mean degree and same degree distribution as the real network (e.g., brain network) of interest, if the real network has higher clustering coefficient and similar characteristic path length (i.e., γ = *C*_net_/*C*_ran_ > 1, λ = *L*_net_ /*L*_ran_~ 1, with *C*_net_ and *L*_net_ indicating the clustering coefficient and characteristic path length of the real network, respectively, and *C*_ran_ and *L*_ran_ indicating the clustering coefficient and characteristic path length of the random network, respectively), the real network would be considered as a small world (Watts and Strogatz, 1998). The ratio between the two parameters γ and λ (γ/λ) can be defined as a parameter σ to measure the small-word-ness, with *σ* > 1 indicating that a network exhibits small-world property (Humphries et al., 2006). Moreover, before calculating the small-world-ness, it is recommended to first evaluate whether small word property of a network is estimable, which is indicated when the mean degree (k) of the network is larger than the log of the number (n) of the network’s nodes (i.e., *k* > log(n); Watts and Strogatz, 1998). Note that small-world property tends to be not estimable for higher thresholds. This is because as the threshold increases, more weak connections will be removed from the network and the network will become sparser. As a result, the mean degree will decrease and is more likely to be less than the log of the number of the network’s nodes (Watts and Strogatz, 1998; Achard et al., 2006; He et al., 2007). In the present study investigating brain networks, for each of the whole brain network and the 10 predefined networks, following the procedure described by Jäncke and Langer (2011) and Langer et al. (2012), the correlation matrices of all participants were first averaged to obtain an average correlation matrix and the corresponding adjacency matrix was calculated. Then for each threshold, whether small word property of a network is estimable was evaluated, and the small-word-ness (σ) was calculated. Note that the following analyses only included the thresholds at which small word property of a network was estimable (i.e., *k* > log(n)) and existed (*σ* > 1). After that, global efficiency was calculated for each network at each threshold for each participant, which was mathematically expressed as the inverse of the average shortest path length in the network (Wang et al., 2010; Beaty et al., 2016).

### Behavioral Assessment

Participants completed a Chinese version of the ERQ (Gross and John, 2003; Wang et al., 2007). The Chinese version of ERQ has satisfactory internal consistency and test-retest reliability (Wang et al., 2007). The ERQ had 10 items, with four items measuring ES and the other six items measuring CR. An example item for ES was: “I control my emotions by not expressing them”; and an example item for CR was “When I want to feel more positive emotion (such as joy or amusement), I change what I’m thinking about”. Participants answered to these items on a 1-to-7 Linkert scale (“1” = “strongly disagree”; “7” = “strongly agree”), to indicate their habitual use of these two ER strategies. Participants further completed a Chinese version of negative affect (NA) subscale of the Positive and NA Schedule (PANAS; Watson et al., 1988; Qiu et al., 2008). The Chinese version of NA subscale had nine items, and participants indicated their general frequency of negative mood states on a 1-to-5 Likert scale (“1” = “not at all”, “5” = “extremely”).

### Structural Equation Modeling (SEM)

**Figure 1:**
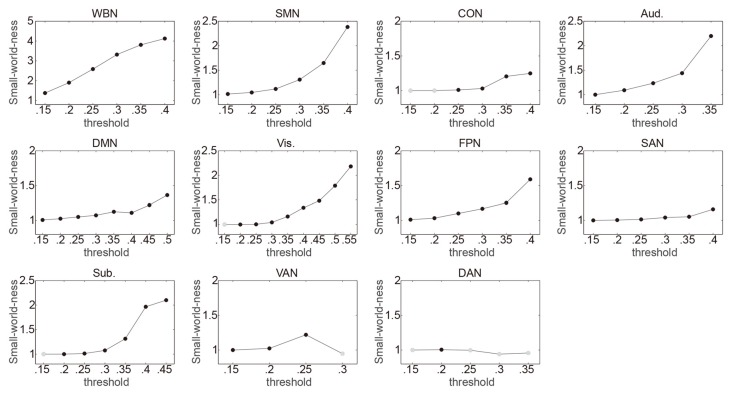
Illustrating the small-world-ness of networks under different thresholds. Whole brain network (WBN), somato-motor network (SMN), cingulo-opercular network (CON), auditory network (Aud.), default mode network (DMN), visual network (Vis.), frontoparietal network (FPN), salience network (SAN), subcortical network (Sub.), ventral attention network (VAN) and dorsal attention network (DAN) represent the WBN, SMN, CON, Aud., DMN, Vis., FPN, SAN, Sub., VAN and DAN, respectively. For each threshold (Fisher transformed *r* ranging from 0.15 to 0.85 in 0.05 steps; totally 15 thresholds), it was first evaluated whether small word property of a network was estimable. The thresholds at which small word property of the network was not estimable were not included in following analyses and are not presented in the figure. The small-word-ness (σ) was then calculated with *σ* > 1 indicating that a network exhibits small-world property. The thresholds at which *σ* > 1 are indicated by black circles, and the thresholds at which *σ* ≤ 1 are indicated by gray circles and were not included in following analyses.

The relationship between trait measures of ER (ES/CR) and global efficiency of a network was examined using SEM, which used latent variables to model error variance separately from true measurement variance (Skrondal and Rabe-Hesketh, 2004; Beaty et al., 2016; Figure 1A). ES and CR were formed as latent variables by specifying their corresponding questionnaire items (four items for ES and six items for CR) as indicators. Age and gender were modeled as observed variables. Note that in order to control for NA, the NA score was also modeled as an observed variable (Giuliani et al., 2011a,b). The analyses without controlling for the NA score are presented in the Supplementary Materials (see “Analyses without modeling the NA score as an observed variable”, Supplementary Tables S1, S2). Standardized regression coefficients were reported. SEM analyses were conducted with Mplus 8 using maximum likelihood robust estimation.

## Results

The mean scores of the two subscales of ERQ were: 2.92 (SD = 1.02) for ES and 4.99 (SD = 0.89) for CR. Consistent with previous results, the ES and CR scores did not statistically correlate with each other (*r* = 0.037, *p* > 0.05), and the internal consistency was acceptable (Cronbach’s α was 0.73 and 0.86 for ES and CR, respectively; Gross and John, 2003; Kühn et al., 2011; Picó-Pérez et al., 2017). The mean score of the NA subscale was 19.88 (SD = 5.28). Consistent with previous reports (Giuliani et al., 2011a,b), the NA score did not correlate with the ES score (*r* = 0.14, *p* = 0.32) or the CR score (*r* = 0.018, *p* = 0.90), and no NA outlier (>3 SD) was found.

The relationship between ES/CR and global efficiency of each of the whole brain network and the 10 networks was examined using SEM over the 15 network thresholds. In sum, the data fit the model well regarding the model fit indices, e.g., comparative fit index (CFI), Tucker–Lewis index (TLI), root mean square error of approximation (RMSEA) and standardized root mean square residual (SRMR; Hu and Bentler, 1998; Brown, 2006; see “Supplementary description of SEM” in the Supplementary Materials for further details of the SEM method), providing support for the proposed theoretical models. Whether a network would have small world property was first evaluated for each threshold, and the following analyses only included the thresholds at which small word property of the network was estimable (i.e., *k* > log(n)) and was presented (*σ* > 1; see the “Materials and Methods” section for details). In general, consistent with previous findings (Watts and Strogatz, 1998; Achard et al., 2006; He et al., 2007), networks lacked small word property for higher thresholds (Figure 1). Connection density was assessed and listed in Table 1. Global efficiency distributions under different thresholds are listed for all networks in Supplementary Figure S1.

**Table 1:** Connection density.

Threshold	WBN (264)	SMN (35)	CON (14)	Aud. (13)	DMN (58)	Vis. (31)	FPN (25)	SAN (18)	Sub. (13)	VAN (9)	DAN (11)
0.15	52.97	67.67	N\A	70.25	72.10	N\A	72.10	73.76	N\A	70.66	N\A
0.2	40.60	57.90	N\A	59.56	63.06	75.23	63.03	64.50	78.90	61.81	65.19
0.25	30.43	49.04	61.77	50.40	54.42	68.01	54.40	55.07	70.14	51.56	N\A
0.3	22.40	41.11	53.64	41.03	46.13	60.77	46.02	46.53	60.92	N\A	N\A
0.35	16.25	34.07	45.26	33.31	38.59	53.04	38.30	38.45	51.47	N\A	N\A
0.4	11.61	27.80	37.29	N\A	31.53	45.30	30.85	31.37	42.52	N\A	N\A
0.45	N\A	N\A	N\A	N\A	25.24	37.86	N\A	N\A	33.79	N\A	N\A
0.5	N\A	N\A	N\A	N\A	19.93	31.25	N\A	N\A	N\A	N\A	N\A
0.55	N\A	N\A	N\A	N\A	N\A	25.66	N\A	N\A	N\A	N\A	N\A
0.6	N\A	N\A	N\A	N\A	N\A	N\A	N\A	N\A	N\A	N\A	N\A
0.65	N\A	N\A	N\A	N\A	N\A	N\A	N\A	N\A	N\A	N\A	N\A
0.7	N\A	N\A	N\A	N\A	N\A	N\A	N\A	N\A	N\A	N\A	N\A
0.75	N\A	N\A	N\A	N\A	N\A	N\A	N\A	N\A	N\A	N\A	N\A
0.8	N\A	N\A	N\A	N\A	N\A	N\A	N\A	N\A	N\A	N\A	N\A
0.85	N\A	N\A	N\A	N\A	N\A	N\A	N\A	N\A	N\A	N\A	N\A

*Average connection density (%; i.e., actual number of edges in a graph relative to the total number of possible edges) across participants is listed for all networks at all thresholds (Fisher transformed *r*). The number of regions in a network is indicated below each network name. Note that the thresholds at which small word property of a network was not estimable and was not presented were not included in the following analyses and are marked by N\A (see Figure 1). Other conventions are as in Figure 1*.

**Figure 2:**
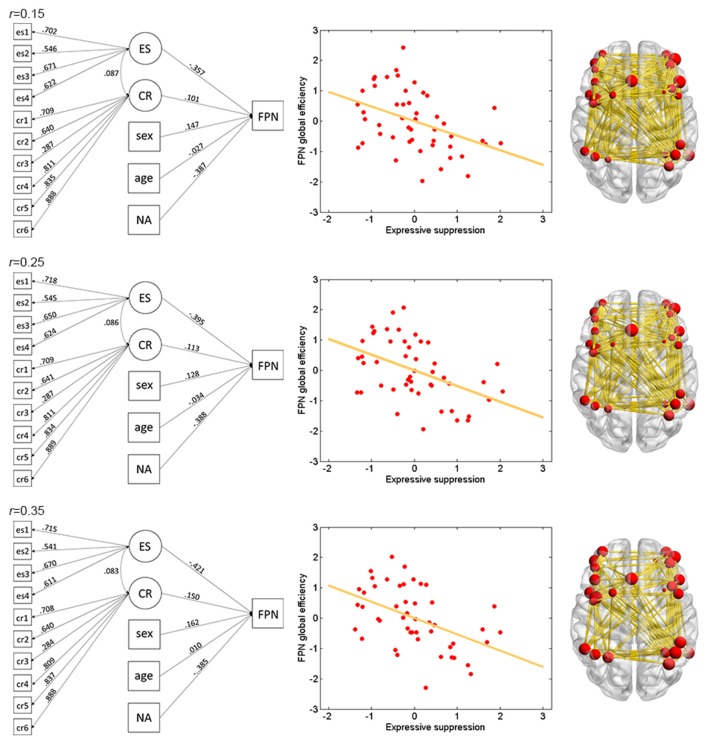
Illustration of association between expressive suppression (ES) and global efficiency in the FPN. Shown are the results for thresholds 0.15 (top row), 0.25 (middle row) and 0.35 (bottom row). For each threshold, structural equation modeling (SEM) is presented on the left, which shows effects of the latent emotion regulation (ER) variables on FPN efficiency. es1–4 refer to the four questionnaire items for ES and cr1–6 refer to the six questionnaire items for cognitive reappraisal (CR). The paths are standardized coefficients. NA indicates negative affect. Scatter plot of the association between latent ES and FPN efficiency is presented in the middle. Note that all variables were standardized by Z-transformation. Nodes and edges that were used to define FPN are presented on the right for a representative participant (the size of nodes represents the degree). Other conventions are as in Table 2.

**Figure 3:**
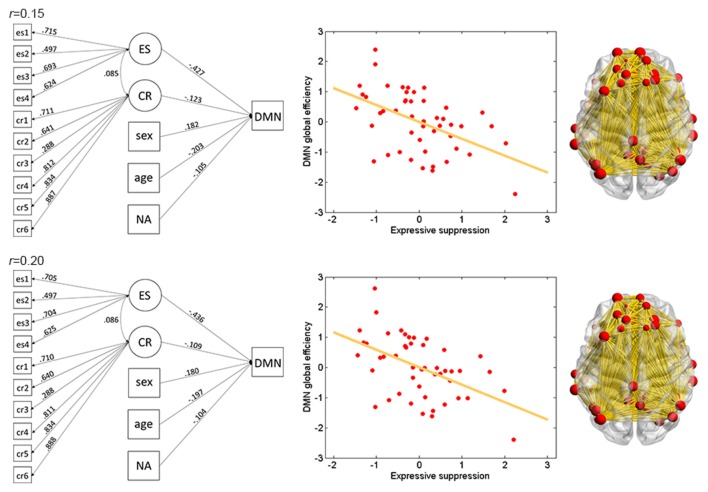
Illustration of association between ES and global efficiency in the DMN. Results for thresholds 0.15 (top row) and 0.2 (bottom row) are shown. Other conventions are as in Figure 2.

The results of ES are listed in Table 2. Note that it has been suggested to examine network properties over a wide range of thresholds since currently there is no “good” threshold for graphic analysis, and findings observed across a range of thresholds (in contrast to those occasionally observed at only a single or a few thresholds) would be considered more reliable (Stam et al., 2006; Bullmore and Sporns, 2009; van den Heuvel et al., 2009; Langer et al., 2012; Power et al., 2013; Xu et al., 2015). In the present results the most reliable association was found between ES and FPN, which was statistically significant (*p*_corrected_ < 0.05, corrected for 10 networks using Bonferroni correction) for thresholds from 0.15 to 0.4 (although marginally significant at threshold 0.2; Table 2 and Figure 2). The association between ES and DMN would also be considered reliable, which was statistically significant (*p*_corrected_ < 0.05) for thresholds 0.15 and 0.2 and was marginally significant for thresholds 0.25 and 0.3 (Table 2 and Figure 3). Moreover, ES was statistically (*p*_corrected_ < 0.05) associated with Aud. at threshold 0.15 (the association was marginally significant for thresholds 0.2 and 0.3) and was marginally associated with the whole brain network at thresholds 0.15, 0.2 and 0.25; which may not be as reliable as the associations between ES and FPN and between ES and DMN.

**Table 2:** Association between expressive suppression (ES) and network global efficiency.

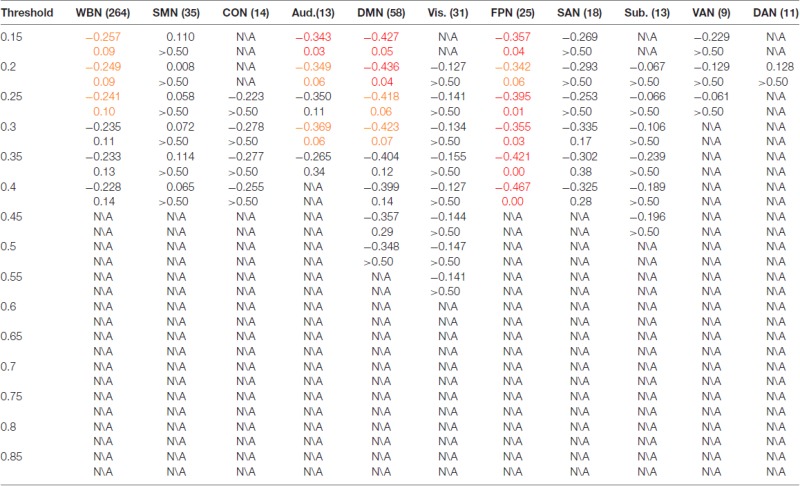

*For each network at each threshold, association *β* value (up row in a data cell) and corrected *p* value (Bonferroni corrected for the 10 networks, bottom row in a data cell) are presented. Associations with *p*_corrected_ values ≤ 0.05 are marked with the red color and associations with *p*_corrected_ values ≤ 0.1 are marked with the orange color. Other conventions are as in Table 1*.

The results of CR are listed in Table 3. In contrast to ES results in which reliable associations across thresholds were found for the FPN and DMN (and less reliable associations for the Aud. and the whole brain network), CR was not statistically associated with efficiency in any network at any threshold.

**Table 3:** Association between cognitive reappraisal (CR) and network global efficiency.

Threshold	WBN (264)	SMN (35)	CON (14)	Aud. (13)	DMN (58)	Vis. (31)	FPN (25)	SAN (18)	Sub. (13)	VAN (9)	DAN (11)
0.15	−0.090	−0.236	N\A	−0.225	−0.123	N\A	0.101	0.058	N\A	−0.116	N\A
	>0.50	>0.50	N\A	>0.50	>0.50	N\A	>0.50	>0.50	N\A	>0.50	N\A
0.2	−0.098	−0.021	N\A	−0.185	−0.109	−0.001	0.090	0.077	0.053	−0.125	0.173
	>0.50	>0.50	N\A	>0.50	>0.50	>0.50	>0.50	>0.50	>0.50	>0.50	>0.50
0.25	−0.110	−0.239	0.013	−0.117	−0.134	−0.017	0.113	0.007	0.100	−0.198	N\A
	0.48	0.44	>0.50	>0.50	>0.50	>0.50	>0.50	>0.50	>0.50	>0.50	N\A
0.3	−0.110	−0.276	0.014	−0.064	−0.156	−0.031	0.158	−0.027	0.025	N\A	N\A
	0.47	0.23	>0.50	>0.50	>0.50	>0.50	>0.50	>0.50	>0.50	N\A	N\A
0.35	−0.114	−0.231	−0.004	−0.144	−0.190	−0.041	0.150	0.015	−0.060	N\A	N\A
	0.43	0.48	>0.50	>0.50	>0.50	>0.50	>0.50	>0.50	>0.50	N\A	N\A
0.4	−0.120	−0.271	0.014	N\A	−0.179	−0.043	0.144	0.072	0.022	N\A	N\A
	0.39	0.22	>0.50	N\A	>0.50	>0.50	>0.50	>0.50	>0.50	N\A	N\A
0.45	N\A	N\A	N\A	N\A	−0.217	−0.055	N\A	N\A	−0.056	N\A	N\A
	N\A	N\A	N\A	N\A	0.50	>0.50	N\A	N\A	>0.50	N\A	N\A
0.5	N\A	N\A	N\A	N\A	−0.193	−0.041	N\A	N\A	N\A	N\A	N\A
	N\A	N\A	N\A	N\A	>0.50	>0.50	N\A	N\A	N\A	N\A	N\A
0.55	N\A	N\A	N\A	N\A	N\A	−0.052	N\A	N\A	N\A	N\A	N\A
	N\A	N\A	N\A	N\A	N\A	>0.50	N\A	N\A	N\A	N\A	N\A
0.6	N\A	N\A	N\A	N\A	N\A	N\A	N\A	N\A	N\A	N\A	N\A
	N\A	N\A	N\A	N\A	N\A	N\A	N\A	N\A	N\A	N\A	N\A
0.65	N\A	N\A	N\A	N\A	N\A	N\A	N\A	N\A	N\A	N\A	N\A
	N\A	N\A	N\A	N\A	N\A	N\A	N\A	N\A	N\A	N\A	N\A
0.7	N\A	N\A	N\A	N\A	N\A	N\A	N\A	N\A	N\A	N\A	N\A
	N\A	N\A	N\A	N\A	N\A	N\A	N\A	N\A	N\A	N\A	N\A
0.75	N\A	N\A	N\A	N\A	N\A	N\A	N\A	N\A	N\A	N\A	N\A
	N\A	N\A	N\A	N\A	N\A	N\A	N\A	N\A	N\A	N\A	N\A
0.8	N\A	N\A	N\A	N\A	N\A	N\A	N\A	N\A	N\A	N\A	N\A
	N\A	N\A	N\A	N\A	N\A	N\A	N\A	N\A	N\A	N\A	N\A
0.85	N\A	N\A	N\A	N\A	N\A	N\A	N\A	N\A	N\A	N\A	N\A
	N\A	N\A	N\A	N\A	N\A	N\A	N\A	N\A	N\A	N\A	N\A

*Conventions are as in Table 2*.

## Discussion

The present study examined the relationship between trait measures of ER (ES and CR) and global efficiency in resting-state brain networks (the whole brain network and 10 predefined networks) using SEM over 15 network thresholds (although in general networks lacked small word property for higher thresholds). The results showed that ES was reliably associated with efficiency in the fronto-parietal network (FPN) and default-mode network (DMN).

The FPN includes primarily portions of the frontal cortex and parietal cortex, in which the lateral PFC is a core region (Vincent et al., 2008; Dodds et al., 2011; Cole et al., 2013; Smith et al., 2013). The lateral PFC is an essential brain area for flexible control of thoughts and actions (MacDonald et al., 2000; Kerns et al., 2004; Egner and Hirsch, 2005) and is involved in various cognitive control tasks (for reviews, see Matsumoto and Tanaka, 2004; Badre and D’Esposito, 2009). The FPN has been shown to be related to task control processes (Dodds et al., 2011; Power et al., 2011; Cole et al., 2013, 2014b). The involvement of the lateral PFC in ES as shown in previous studies (Goldin et al., 2008) would be in line with the current finding of the association between ES and efficiency in the FPN. The DMN comprises majorly the media PFC, posterior cingulate cortex (PCC) and inferior parietal cortex (Buckner et al., 2008; Broyd et al., 2009; Power et al., 2011). The DMN was initially observed when participants are not focused on an specific task (e.g., in a resting state) and following studies have suggested that the DMN is related to a wide range of spontaneous and self-generated processes, such as episodic future thinking, autobiographical memory processing, mind wandering and thinking about others (Anticevic et al., 2012; Andrews-Hanna et al., 2014; Fox et al., 2015; Hamilton et al., 2015; Stawarczyk and D’Argembeau, 2015). Note that it has been suggested that the DMN is associated with reflecting about one’s own emotional state, and understanding others’ emotions (Andrews-Hanna, 2012; Andrews-Hanna et al., 2014). As a key region of the DMN, the media PFC has been found to be engaged in ES (Goldin et al., 2008; Welborn et al., 2009), which would be in agreement with the present finding of the association between ES and efficiency in the DMN. Combining these findings, it could be possible that for ES, efficiency in the DMN is related to emotional processing of an incident and efficiency in the FPN is associated with control of emotional responses. Furthermore, it was worth noting that an association between ES and efficiency in the CON was not observed, given dACC volume has been found to be related to ES (Dosenbach et al., 2007; Power et al., 2011; Hermann et al., 2014). This, in line with previous results (Sampaio et al., 2014; Beaty et al., 2016), could indicate that global functioning of a brain network may not be engaged in a cognitive process even when a brain region within the network is related to the process. Moreover, the present study systematically explored the whole brain network and 10 predefined networks and ES showed less reliable association with the Aud., which would require further investigation.

The ERQ assesses the habitual ES and CR usage: the more frequent one utilizes ES/CR to regulate his/her emotions, the higher this person will score on the ES/CR scale; and vice versa (Gross and John, 2003). In previous brain structural studies using the ERQ, positive relationships between ES and brain region volumes were often reported (Giuliani et al., 2011a; Kühn et al., 2011; Hermann et al., 2014; Wang et al., 2017); whereas Welborn et al. (2009) did not observe such positive associations but rather found a negative relationship between the vmPFC volume and ES. It has been suggested that the discrepancies among results of different studies could be due to different methodological factors such as whole-brain vs. ROI analysis (Hermann et al., 2014) and voxel- vs. surface-based analysis (Moore et al., 2016). For the current analyses of global functioning of brain networks, ES was negatively associated with efficiency in the FPN and DMN (the association with the Aud. was less reliable). Global efficiency in the whole brain networks has been found negatively associated with some mental disorders (Wang et al., 2009; Meng et al., 2014; Collin et al., 2016), such as major depressive disorder (Meng et al., 2014); and notably, ES has been consistently connected to negative affective and social consequences, including depressive symptoms (Gross, 2002). This could be one plausible clue for the currently observed negative association between ES and network efficiency.

Moreover, association between CR and efficiency in a network was not observed. While the lack of CR association was unexpected, it appeared to be in agreement with a report showing that CR usage as measured with the ERQ was associated with fewer brain regions as compared to ES usage; while ES was found to be related to multiple brain regions including the vmPFC, dmPFC and dACC, CR was only associated with the amygdala (Hermann et al., 2014). The discrepancies in structural (Hermann et al., 2014) and functional network (as in the present study) properties between ES and CR usages require to be further clarified.

The present study would be considered as a preliminary attempt to investigate the relationship between ER and complex brain networks and has many limitations. Besides the limitations as discussed above, the topological properties of complex networks can be assessed by a wide variety of measures (Bullmore and Sporns, 2009; Rubinov and Sporns, 2010; Wang et al., 2010; De Vico Fallani et al., 2014). While the present study focused on the global metric of global efficiency, the topological properties of complex networks would be investigated comprehensively in future ER studies. In particular, roles of individual nodes (regions) in ER remain to be addressed by using metrics such as centrality. Accordingly, the currently adopted 10 predefined brain networks were defined based on 264 putative brain regions. While the 264-region parcellation and the 10 networks are helpful for investigation of network properties in general, investigation of emotion-related functions would require further work. Particularly, an emotion-related network is not defined and amygdala -a key area in emotion processing- is not specifically defined (Power et al., 2011; Cole et al., 2013). Future research could define the region of interest (ROI) of the amygdala structurally or functionally (Poldrack, 2007) and define an emotion-related network by calculating correlation between the amygdala ROI with all other voxels in the brain (van den Heuvel and Hulshoff Pol, 2010). Then topological properties of the emotion-related network could be calculated and their relationships with ER could be examined.

In sum, ER is essential for human adaptive functioning (Gross, 1998; Ochsner and Gross, 2005). Whereas previous brain-imaging studies have investigated functions of individual brain areas in ER, the role of global functioning of brain networks remains unknown. The present finding of the association of ES with global efficiency in the FPN and DMN suggests that efficient organization of specific brain networks is a fundamental neural mechanism for ER. Meanwhile, whereas the results of current graphic analyses of resting-state functional networks showed consistency with the regional results from previous task-based fMRI and structural MRI studies (e.g., association between ES and areas in the FPN and DMN), discrepancies in the results were also revealed and would be addressed in future research (e.g., lack of association between ES and efficiency in the CON, lack of association between CR and network efficiency). Therefore, while the present findings suggest the important role of global functioning of brain networks in ER, it is also indicated that combining different research approaches is required for a better understanding of neural mechanisms underlying ER.

## Data Availability

The data generated during and/or analyzed during the current study are available from the corresponding author on reasonable request.

## Author Contributions

LZ, CH, MH and XW designed the research. SZ, LG, JY and YH collected MRI data. LZ, CW, JY and QW analyzed MRI data. CH, XX, QC and HZ collected questionnaire data. CH analyzed questionnaire data. JP and LZ performed SEM analysis. JP, LZ, CH, MH and XW wrote the manuscript. All authors commented on the manuscript.

## Conflict of Interest Statement

The authors declare that the research was conducted in the absence of any commercial or financial relationships that could be construed as a potential conflict of interest.

## Footnotes

^1^http://www.fil.ion.ucl.ac.uk/spm
