# Supplementary material for: Emotion Regulation and Complex Brain Networks: Association Between Expressive Suppression and Efficiency in the Fronto-Parietal Network and Default-Mode Network
Source: Front Hum Neurosci. 2018 Mar 16;12:70. doi: 10.3389/fnhum.2018.00070 (PMC5890121; doi:10.3389/fnhum.2018.00070)
Supplement: Supplementary file 1 [file Data_Sheet_1.pdf]

Title: Emotion regulation and complex brain networks: Association between expressive suppression and efficiency in the fronto-parietal network and default-mode network

Authors: Junhao Pan[#], Liying Zhan[#], ChuanLin Hu[#], Junkai Yang, Cong Wang, Li Gu, Shengqi Zhong, Yingyu Huang, Qian Wu, Xiaolin Xie, Qijin Chen, Hui Zhou, Miner Huang[*], Xiang Wu[*]
(These authors contributed equally to this work)

Supplementary Materials

Fig. S1; Table S1 to S2; Supplementary Text.

Fig. S1. **Illustration of global efficiency distributions under different thresholds.** The distributions (frequency histograms) are presented for all networks. x axis indicates the value of global efficiency, which was not standardized and ranged from 0 to 1. Note that the thresholds at which small word property of a network was not estimable and was not presented were not included in the following analyses and are not plotted in the figure (see Fig. 1). Other conventions are as in Fig.1.

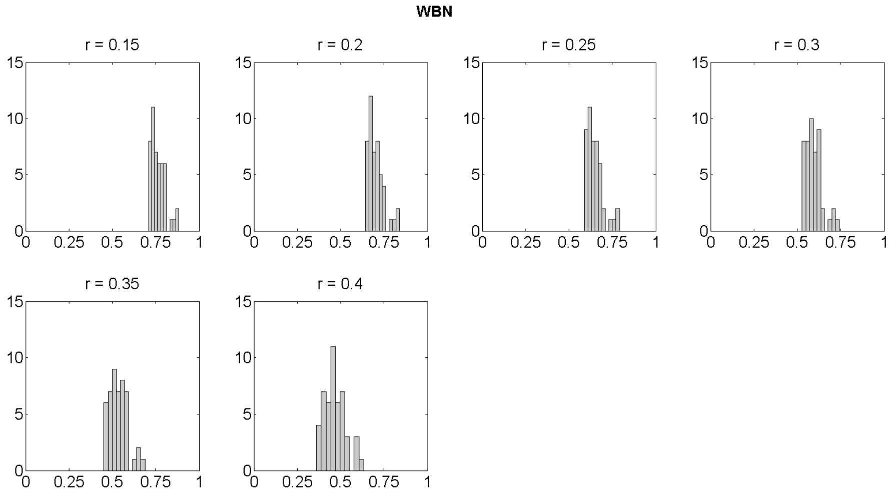

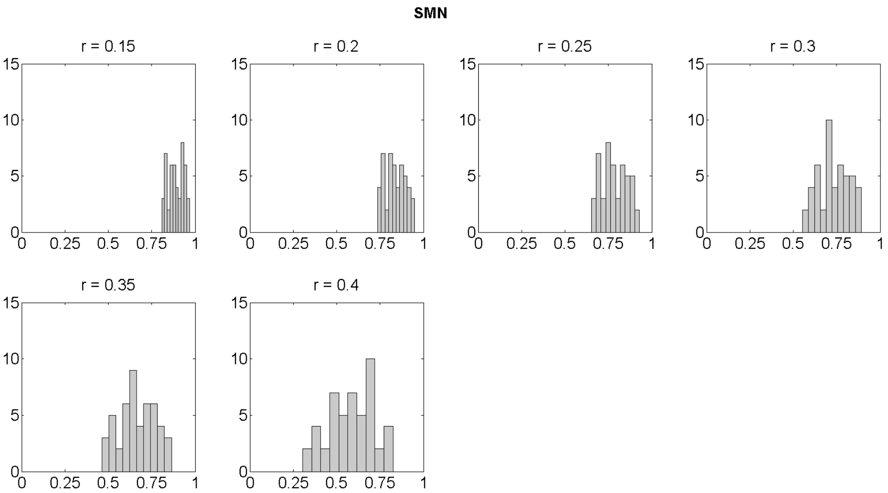
SMN
r = 0.15
r = 0.2
r = 0.25
r = 0.3
r = 0.35
r = 0.4

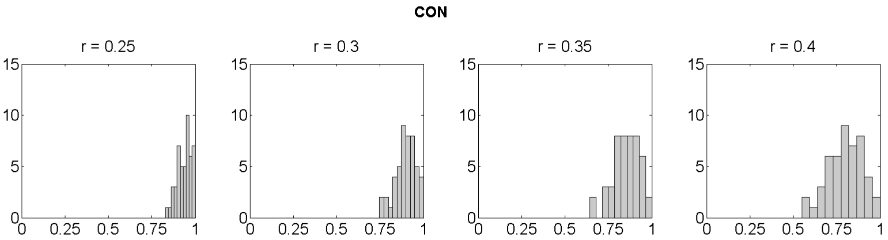
CON
r = 0.25
r = 0.3
r = 0.35
r = 0.4

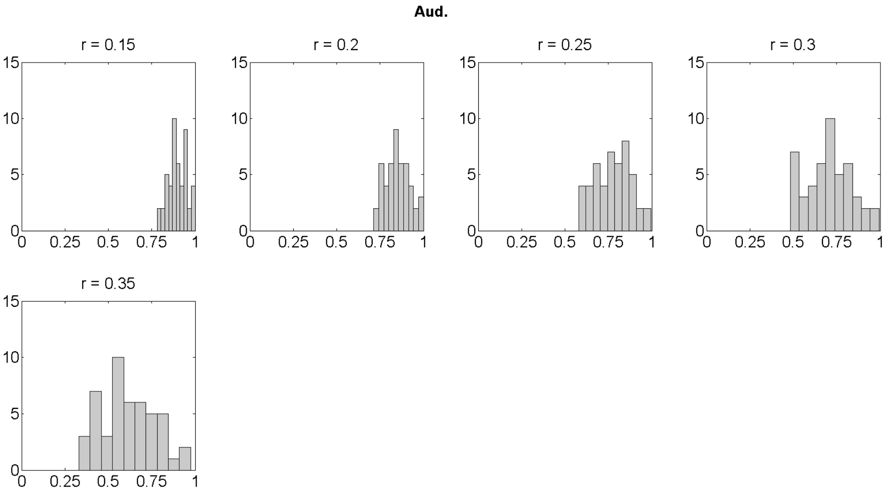
Aud.
r = 0.15
r = 0.2
r = 0.25
r = 0.3
r = 0.35

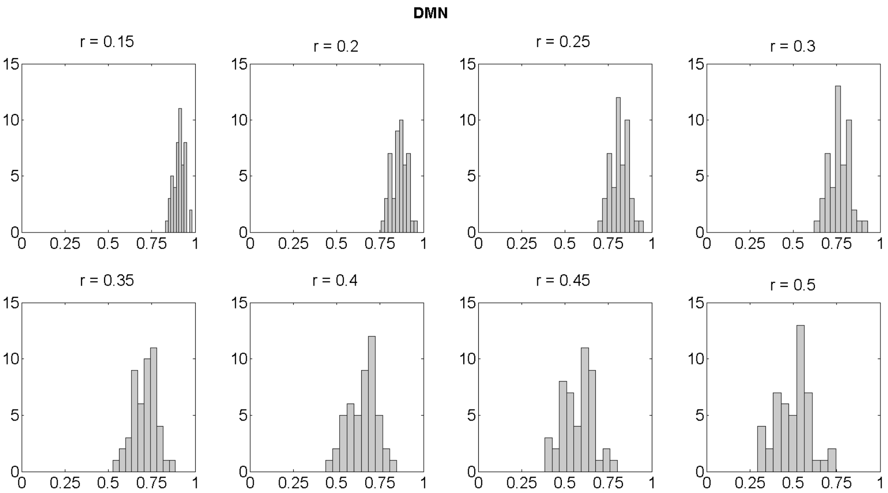
DMN
r = 0.15
r = 0.2
r = 0.25
r = 0.3
r = 0.35
r = 0.4
r = 0.45
r = 0.5

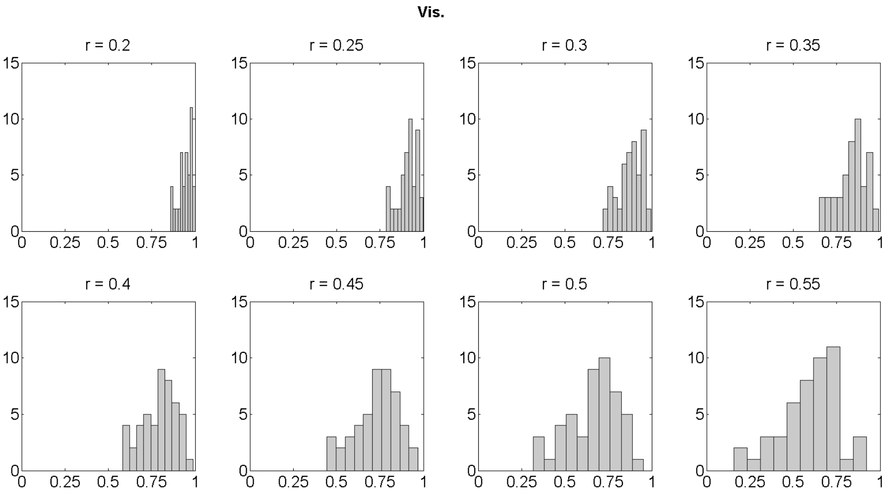
Vis.
r = 0.2
r = 0.25
r = 0.3
r = 0.35
r = 0.4
r = 0.45
r = 0.5
r = 0.55

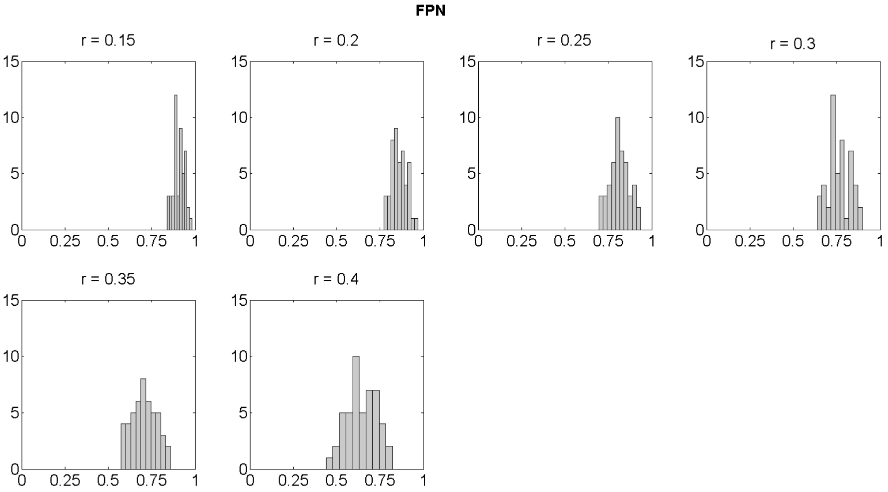
FPN
r = 0.15
r = 0.2
r = 0.25
r = 0.3
r = 0.35
r = 0.4

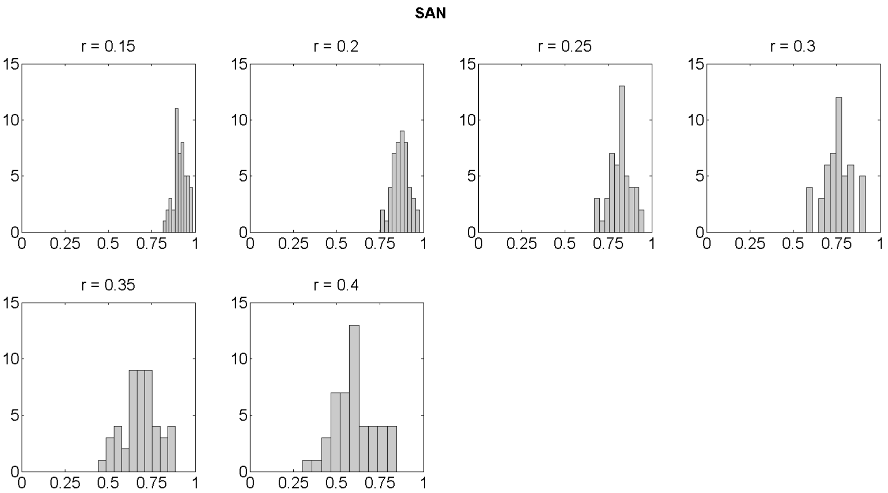
SAN
r = 0.15
r = 0.2
r = 0.25
r = 0.3
r = 0.35
r = 0.4

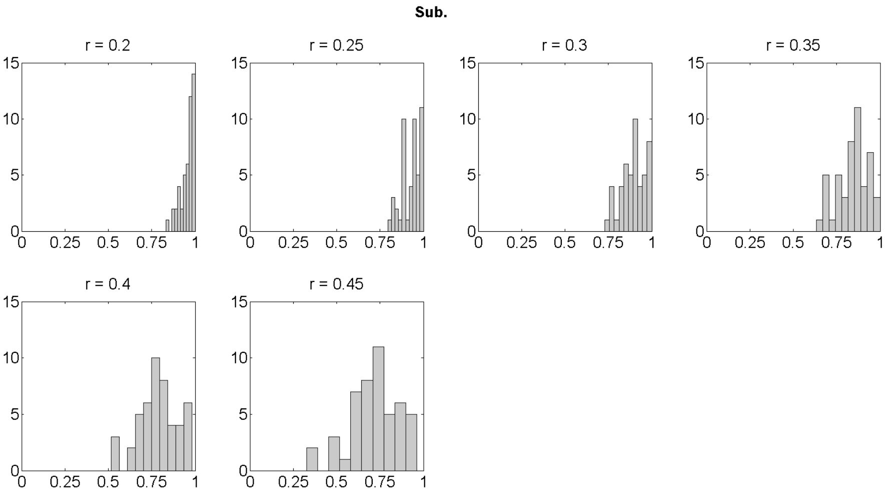
Sub.
r = 0.2
r = 0.25
r = 0.3
r = 0.35
r = 0.4
r = 0.45

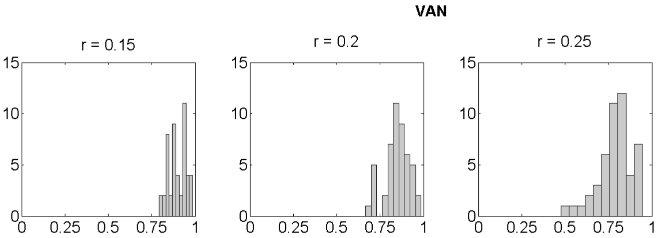
VAN
r = 0.15
r = 0.2
r = 0.25

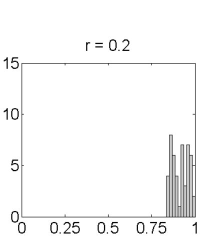
DAN
r = 0.2

Table S1. Association between expressive suppression (ES) and network global efficiency. The NA score was not modeled as an observed variable in the analyses. Other conventions are as in Table 2.

Threshold	WBN (264)	SMN (35)	CON (14)	Aud. (13)	DMN (58)	Vis. (31)	FPN (25)	SAN (18)	Sub. (13)	VAN (9)	DAN (11)
0.15	-.293	.089	N\A	-.332	-.438	N\A	-.415	-.292	N\A	-.278	N\A
	.03	>.50	N\A	.03	.03	N\A	.00	.35	N\A	>.50	N\A
0.2	-.286	.076	N\A	-.344	-.448	-.165	-.409	-.316	-.138	-.195	-.161
	.04	>.50	N\A	.08	.02	>.50	.01	.30	>.50	>.50	>.50
0.25	-.277	.036	-.264	-.346	-.430	-.185	-.452	-.293	-.135	-.096	N\A
	.04	>.50	>.50	.11	.03	>.50	.00	>.50	>.50	>.50	N\A
0.3	-0.273	.054	-.320	-.382	-.437	-.178	-.417	-.373	-.177	N\A	N\A
	.04	>.50	>.50	.04	.04	>.50	.00	.05	>.50	N\A	N\A
0.35	-0.276	.094	-.277	-.277	-.431	-.200	-.478	-.351	-.295	N\A	N\A
	.04	>.50	>.50	.30	.03	>.50	.00	.05	>.50	N\A	N\A
0.4	-0.272	.049	-.295	N\A	-.418	-.160	-.521	-.375	-.252	N\A	N\A
	.05	>.50	>.50	N\A	.07	>.50	.00	.04	>.50	N\A	N\A
0.45	N\A	N\A	N\A	N\A	-.378	-.175	N\A	N\A	-.263	N\A	N\A
	N\A	N\A	N\A	N\A	.13	>.50	N\A	N\A	>.50	N\A	N\A
0.5	N\A	N\A	N\A	N\A	-.370	-.178	N\A	N\A	N\A	N\A	N\A
	N\A	N\A	N\A	N\A	.23	>.50	N\A	N\A	N\A	N\A	N\A
0.55	N\A	N\A	N\A	N\A	N\A	-.174	N\A	N\A	N\A	N\A	N\A
	N\A	N\A	N\A	N\A	N\A	>.50	N\A	N\A	N\A	N\A	N\A
0.6	N\A	N\A	N\A	N\A	N\A	N\A	N\A	N\A	N\A	N\A	N\A
	N\A	N\A	N\A	N\A	N\A	N\A	N\A	N\A	N\A	N\A	N\A
0.65	N\A	N\A	N\A	N\A	N\A	N\A	N\A	N\A	N\A	N\A	N\A
	N\A	N\A	N\A	N\A	N\A	N\A	N\A	N\A	N\A	N\A	N\A
0.7	N\A	N\A	N\A	N\A	N\A	N\A	N\A	N\A	N\A	N\A	N\A
	N\A	N\A	N\A	N\A	N\A	N\A	N\A	N\A	N\A	N\A	N\A
0.75	N\A	N\A	N\A	N\A	N\A	N\A	N\A	N\A	N\A	N\A	N\A
	N\A	N\A	N\A	N\A	N\A	N\A	N\A	N\A	N\A	N\A	N\A
0.8	N\A	N\A	N\A	N\A	N\A	N\A	N\A	N\A	N\A	N\A	N\A
	N\A	N\A	N\A	N\A	N\A	N\A	N\A	N\A	N\A	N\A	N\A
0.85	N\A	N\A	N\A	N\A	N\A	N\A	N\A	N\A	N\A	N\A	N\A
	N\A	N\A	N\A	N\A	N\A	N\A	N\A	N\A	N\A	N\A	N\A

Table S2. Association between cognitive reappraisal (CR) and network global efficiency. The NA score was not modeled as an observed variable in the analyses. Other conventions are as in Table 3.

Threshold	WBN (264)	SMN (35)	CON (14)	Aud. (13)	DMN (58)	Vis. (31)	FPN (25)	SAN (18)	Sub. (13)	VAN (9)	DAN (11)
0.15	-.070<br>>.50	-.197<br>>.50	N\A<br>N\A	-.209<br>>.50	-.126<br>>.50	N\A<br>N\A	.048<br>>.50	.111<br>>.50	N\A<br>N\A	-.092<br>>.50	N\A<br>N\A
0.2	-.078<br>>.50	-.198<br>>.50	N\A<br>N\A	-.177<br>>.50	-.107<br>>.50	.003<br>>.50	.035<br>>.50	.154<br>>.50	.098<br>>.50	-.106<br>>.50	.195<br>>.50
0.25	-.091<br>>.50	-.201<br>>.50	.018<br>>.50	-.112<br>>.50	-.132<br>>.50	-.008<br>>.50	.058<br>>.50	.077<br>>.50	.136<br>>.50	-.184<br>>.50	N\A<br>N\A
0.3	-.091<br>>.50	-.244<br>>.50	.031<br>>.50	-.048<br>>.50	-.159<br>>.50	-.023<br>>.50	.090<br>>.50	.030<br>>.50	.009<br>>.50	N\A<br>N\A	N\A<br>N\A
0.35	-.103<br>>.50	-.210<br>>.50	.003<br>>.50	-.126<br>>.50	-.210<br>.46	-.036<br>>.50	.081<br>>.50	.060<br>>.50	-.044<br>>.50	N\A<br>N\A	N\A<br>N\A
0.4	-.112<br>>.50	-.243<br>>.50	.012<br>>.50	N\A<br>N\A	-.179<br>>.50	-.026<br>>.50	.117<br>>.50	.123<br>>.50	.036<br>>.50	N\A<br>N\A	N\A<br>N\A
0.45	N\A<br>N\A	N\A<br>N\A	N\A<br>N\A	N\A<br>N\A	-.224<br>.41.	-.028<br>>.50	N\A<br>N\A	N\A<br>N\A	-.046<br>>.50	N\A<br>N\A	N\A<br>N\A
0.5	N\A<br>N\A	N\A<br>N\A	N\A<br>N\A	N\A<br>N\A	-.208<br>>.50	-.020<br>>.50	N\A<br>N\A	N\A<br>N\A	N\A<br>N\A	N\A<br>N\A	N\A<br>N\A
0.55	N\A<br>N\A	N\A<br>N\A	N\A<br>N\A	N\A<br>N\A	N\A<br>N\A	-.032<br>>.50	N\A<br>N\A	N\A<br>N\A	N\A<br>N\A	N\A<br>N\A	N\A<br>N\A
0.6	N\A<br>N\A	N\A<br>N\A	N\A<br>N\A	N\A<br>N\A	N\A<br>N\A	N\A<br>N\A	N\A<br>N\A	N\A<br>N\A	N\A<br>N\A	N\A<br>N\A	N\A<br>N\A
0.65	N\A<br>N\A	N\A<br>N\A	N\A<br>N\A	N\A<br>N\A	N\A<br>N\A	N\A<br>N\A	N\A<br>N\A	N\A<br>N\A	N\A<br>N\A	N\A<br>N\A	N\A<br>N\A
0.7	N\A<br>N\A	N\A<br>N\A	N\A<br>N\A	N\A<br>N\A	N\A<br>N\A	N\A<br>N\A	N\A<br>N\A	N\A<br>N\A	N\A<br>N\A	N\A<br>N\A	N\A<br>N\A
0.75	N\A<br>N\A	N\A<br>N\A	N\A<br>N\A	N\A<br>N\A	N\A<br>N\A	N\A<br>N\A	N\A<br>N\A	N\A<br>N\A	N\A<br>N\A	N\A<br>N\A	N\A<br>N\A
0.8	N\A<br>N\A	N\A<br>N\A	N\A<br>N\A	N\A<br>N\A	N\A<br>N\A	N\A<br>N\A	N\A<br>N\A	N\A<br>N\A	N\A<br>N\A	N\A<br>N\A	N\A<br>N\A
0.85	N\A<br>N\A	N\A<br>N\A	N\A<br>N\A	N\A<br>N\A	N\A<br>N\A	N\A<br>N\A	N\A<br>N\A	N\A<br>N\A	N\A<br>N\A	N\A<br>N\A	N\A<br>N\A

Supplementary Text

Supplementary description of SEM

Structural equation model (SEM) is a powerful and flexible method for modeling multivariate data by a few unobserved latent variables. This statistical method is very popular in educational, psychological, behavioral, and biomedical research (Lee & Song, 2012). Recently, it has also received a great deal attention in neuroimaging for brain effectual connectivity analysis (Ahmad et al., 2016; Beaty et al., 2016; Gates et al., 2011).

In general, SEM consists of two major components. The first component is called measurement model, which is basically a confirmatory factor analysis model. In this study, the measurement model described the relationship between the questionnaire items (indicators) and the latent variable (expressive suppression and cognitive reappraisal).

The second component is called structural model, which examines the inter-relationship among latent variables and other observed variables. In this study, the structural model was used to describe the relationship between emotion regulation (expressive suppression and cognitive reappraisal) and global efficiency of a network, while controlling for age, gender, and the NA score.

The goodness of fit of SEM model was usually evaluated using the following indices: comparative fit index (CFI; Bentler, 1990), Tucker–Lewis index (TLI; Tucker & Lewis, 1973), root mean square error of approximation (RMSEA; Steiger, 1980) and standardized root mean square residual (SRMR; Browne & Cudeck, 1992). The values of CFI and TLI should be larger than 0.90 whereas the value of RMSEA should be less than 0.08, which indicate adequate model fit (Brown, 2006). SRMR with values less than 0.08 is considered adequate (Hu & Bentler, 1998). It is emphasized that the use of multiple and complementary indices is highly recommended (Fan & Sivo, 2005). These above-mentioned criteria are considered only as guidelines, because values of these indices have been found to fluctuate as a function of modeling conditions, and thus the values that are slightly out of the ranges indicated above can still be considered acceptable (Hu & Bentler, 1998; Brown, 2006).

Analyses without modeling the NA score as an observed variable

In the analyses presented in the main text, the score of the negative affect (NA) subscale was modeled as an observed variable. Here the analyses without modeling the NA score as an observed variable are presented (other analysis settings were the same as the analyses in the main text).

The results of ES are listed in Table S1. Reliable association was found between ES and FPN, which was statistically significant ($p_{corrected}<.05$, corrected for 10 networks using Bonferroni correction) for thresholds from 0.15 to 0.4; between ES and DMN, which was statistically significant for thresholds from 0.15 to 0.35 and was marginally significant for threshold 0.4; and between ES and the whole brain network, which was statistically significant for thresholds from 0.15 to 0.4. Moreover, ES was statistically ($p_{corrected}<.05$) associated with Aud. at thresholds 0.15 and 0.3 (the association was marginally significant for threshold 0.2), and was statistically ($p_{corrected}<.05$) associated with SAN at thresholds 0.3, 0.35, and 0.4; which may not be as reliable as the associations between ES and FPN, between ES and DMN, and between ES and the whole brain network. The results of CR are listed in Table S2. CR was not statistically associated with efficiency in any network at any threshold.

The results presented here were generally consistent with the results presented in the main text, except that reliable association between ES and the whole brain network (which was marginally significant for thresholds 0.15, 0.2, and 0.3 in the main text) and less reliable association between ES and the SAN were observed. Importantly, reliable association was consistently found between ES and FPN and between ES and DMN

regardless whether the NA score was modeled as an observed variable, suggesting the validity of the present finding.

References

Ahmad, F., Ahmad, I., Nisa, Z., & Mahmood Ramay, S. (2016). Exploration of connectivity with SEM: An fMRI study of resting state. *International Journal of Imaging Systems and Technology*, *26*(4), 264–269.

Beaty, R. E., Kaufman, S. B., Benedek, M., Jung, R. E., Kenett, Y. N., Jauk, E., … Silvia, P. J. (2016). Personality and complex brain networks: The role of openness to experience in default network efficiency: Openness and the Default Network. *Human Brain Mapping*, *37*(2), 773–779. doi: 10.1002/hbm.23065

Bentler, P. (1990). Comparative fit indexes in structural models. *Psychological Bulletin*, *107*(2), 238—246. doi: 10.1037/0033-2909.107.2.238

Brown, T. A. (2006). *Confirmatory factor analysis for applied research*. New York: Guilford Press.

Browne, M. W., & Cudeck, R. (1992). Alternative Ways of Assessing Model Fit. *Sociological Methods & Research*, *21*(2), 230–258. doi: 10.1177/0049124192021002005

Fan, X., & Sivo, S. A. (2005). Sensitivity of Fit Indexes to Misspecified Structural or Measurement Model Components: Rationale of Two-Index Strategy Revisited. *Structural Equation Modeling: A Multidisciplinary Journal*, *12*(3), 343–367. doi: 10.1207/s15328007sem1203_1

Gates, K. M., Molenaar, P. C. M., Hillary, F. G., & Slobounov, S. (2011). Extended unified SEM approach for modeling event-related fMRI data. *NeuroImage*, *54*(2), 1151–1158. doi: 10.1016/j.neuroimage.2010.08.051

Hu, L., & Bentler, P. M. (1998). Fit indices in covariance structure modeling: Sensitivity to underparameterized model misspecification. *Psychological Methods*, *3*(4), 424.

Lee, S.-Y., & Song, X.-Y. (2012). *Basic and advanced Bayesian structural equation modeling: With applications in the medical and behavioral sciences*. John Wiley & Sons.

Steiger, J. H. (1980). Statistically based tests for the number of common factors. Presented at the Paper presented at the annual meeting of the Psychometric Society, Iowa City, IA, May 1980.

Tucker, L. R., & Lewis, C. (1973). A reliability coefficient for maximum likelihood factor analysis. Psychometrika, 38(1), 1–10. doi: 10.1007/BF02291170
